# Prenatal Exposure to Nicotine in Pregnant Rat Increased Inflammatory Marker in Newborn Rat

**DOI:** 10.1155/2014/274048

**Published:** 2014-08-28

**Authors:** Yosouf Mohsenzadeh, Asghar Rahmani, Javad Cheraghi, Maryam Pyrani, Khairollah Asadollahi

**Affiliations:** ^1^Department of Cardiology, Faculty of Medicine, Ilam University of Medical Sciences, Ilam 6931435647, Iran; ^2^Student Researches Committee, Ilam University of Medical Sciences, Ilam 6931435647, Iran; ^3^Department of Physiology, Faculty of Veterinary, Ilam University, Ilam 6931435647, Iran; ^4^Department of Animal Physiology, Faculty of Veterinary, University of Tehran, Tehran 1419963111, Iran; ^5^Department of Epidemiology, Faculty of Medicine, Ilam University of Medical Sciences, Ilam 6931435647, Iran; ^6^The researches Centre of Psychosocial Injuries, Ilam University of Medical Sciences, Ilam 6931435647, Iran

## Abstract

This study aimed to investigate any inflammatory effect of nicotine on rat embryo by exposing their mothers to different dosages of nicotine during pregnancy. During this experimental study, 32 pregnant healthy Wistar rats were divided into 4 equal groups, including a control and 3 nicotine exposure groups. Injections were performed subcutaneously starting at the first day of pregnancy until parturition. As the dosages of nicotine were increased, the weight gain by pregnant rats and the mean weight of their newborns were significantly reduced. Mean ± SD of hs-CRP was significantly higher among groups exposed to various dosages of nicotine (2, 4, and 6 mg/kg) compared to the control group (*P* < 0.0001) and its increasing rate was also dose dependent. Mean ± SD serum level of IL-6 and TNF-*α* among all groups exposed to nicotine, except for 2 mg/kg nicotine injected group, was increased significantly (*P* < 0.0001). Mean ± SD of serum level of TGF-*β* and nitrite oxide among exposure groups showed significant differences compared to the control group only at the dosage of 6 mg/kg (*P* < 0.0001). The current study showed that exposing pregnant rats to nicotine causes a dose dependent increase in the rate of all the studied inflammatory serum markers among their newborns.

## 1. Introduction

Smoking during pregnancy is one the most important problems in public health globally that causes different harmful outcomes such as intrauterine growth retardation, cardiovascular diseases, and abortion in fetus accompanied with some complications in mothers [1, 2]. The majority of cardiovascular complications due to smoking during pregnancy are attributed to nicotine and previous studies have reported that smoking during pregnancy can cause an alteration in vascular activity in adolescence period. It also causes a reduction in cardiac ability for rehabilitation after ischemia among rats [3, 4]. Nicotine passes from placenta and enters in fetus circulation and causes an increase in vascular resistance and vasopressin that result in umbilical vasoconstriction [5, 6]. Prevalence of prenatal smoking is estimated as 11–30% [7]. There are different studies in the literature that report congenital deficiency and behavioral disorders due to smoking during pregnancy such as hyperactivity, conductive disorder, nicotine dependency, and drug abuse [8–14]. Prenatal exposure to nicotine causes a reduction in explanation of PKC_E_ protein which is a notable factor for adolescence cardiovascular disorders [15]. Some other cardiovascular abnormalities such as variations of heart beats, fetus hypoxia, reduction of antioxidant enzyme of myocardium, and creation of lipid peroxidation have been attributed to prenatal nicotine by different studies [16–18]. Due to lack of information about exposure to nicotine during pregnancy and its effects on inflammatory serologic markers such as nitrite oxide, TGF-*β*, TNF-*α*, IL-6, and CRP among newborns, the present study was carried out to investigate this phenomenon experimentally in rats. The present study was launched to detect the inflammatory effects of nicotine on rat embryo by exposing their mothers to different dosages of nicotine during pregnancy.

## 2. Materials and Methods

### 2.1. Animals

By an experimental study, 32 healthy Wistar rats (weights 150–180 gr), prepared by Pastor Institution in Iran and kept at the same conditions including environmental dampness (38% ± 2), light (12 hours darkness and 12 hours lightness intermittently), temperature (22 ± 2°), and free access to food and water, were investigated. Each 2 virgin female rats were located in a cage with one male rat for 24 hours and in the next morning vaginal smears were examined andif sperm was found in the vaginal smear and vaginal plugs existed, it was considered that they had mated [19]. After mating, the male rat was removed and each female was placed in an individual cage until the end of study. All such pregnant rats were categorised into 4 groups randomly. This research was conducted in accordance with the Principles of Laboratory Animal Care (NIH publication, revised in 1985) and was approved prospectively by Ethics Committee of Ilam University of Medical Sciences, Iran.

### 2.2. Nicotine Exposure

Totally 32 pregnant rats were divided into 4 equal groups including the following: a control group (receiving NaCl 9%, 10 mg/kg/day) and 3 exposure groups of nicotine tartrate (receiving 2 mg/kg/day, 4 mg/kg/day and 6 mg/kg/day of solution (containing (-) nicotine, 56.1 mg/mL bacteriostatic saline, Sigma Co.). Rats were anesthetized by inhalation of 1–3% isoflurane in oxygen and prepared with Alzet osmotic minipumps (Model 2ML4, Du-rect, Cupertino, CA) placed subcutaneously (back of the animal parallel to the spine). Nicotine was infused via osmotic minipumps, starting at the first day of pregnancy until parturition. Pumps were filled with either NaCl 0.9% (sterile saline) or nicotine salt solution. The concentration of the nicotine salt solution was adjusted according to animal's body weight, resulting in a delivery of 2, 4, and 6 mg/kg/day, levels approximately equivalent to those that occur in mild and moderate to heavy smokers [2]. Also injected dosages of nicotine and physiological saline (NaCl 9%), in 4 groups, were calculated in keeping with the weight of the pregnant rats every day.

### 2.3. Investigation of Serum Markers

A blood sample from each rat's newborn was taken from inferior vena cava and was centrifuged at 4000 ×g for 15 minutes in +4°C (temperature rate during centrifuge) and its separated serum was then kept at −70°C (temperature rate until the end of study). Serum hs-CRP, IL-6, TNF-*α*, nitrite oxide, and TGF-*β* were measured using Cusabio Biotech (0.04 ng/mL sensitivity), Diaclone (19 pg/mL sensitivity), Diaclone (20 pg/mL sensitivity), Cusabio Biotech (19.5 pg/mL sensitivity), ABACAM (8 pg/mL sensitivity), and Abacam (19 pg/mL sensitivity) company devices, respectively.

## 3. Statistical Analysis

Data were expressed as mean ± SD. The mean serum levels of hs-CRP, IL-6, TNF-*α*, TGF-*β*, and NO were compared between groups using Kruskal-Wallis or one-way ANOVA, accordingly. All the pairwise multiple comparisons and the comparisons between control and other groups were done using Dunn's nonparametric multiple comparison test.

## 4. Results

The general characteristics of rats, pregnancy, and newborn rats are demonstrated in Table 1. The results showed that as the dosages of nicotine were increased, the weight gains by pregnant rats and the mean weight of their newborns were significantly reduced.

**Figure 1:**
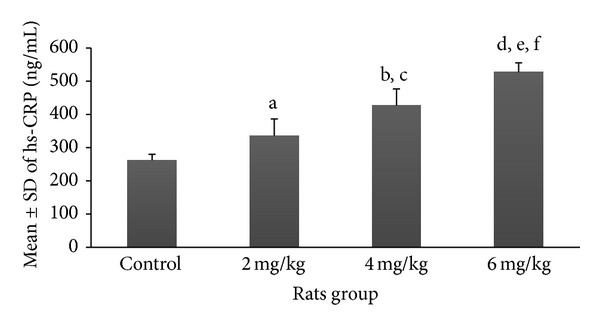
Prenatal exposure to nicotine in pregnant rats increased the inflammatory marker hs-CRP in newborn rats. Mean ± SD of the hs-CRP marker is shown in different groups of newborns. *N* = 8 (a: *P* < 0.05 in 2 mg/kg group versus control group; b and c: *P* < 0.001 in 4 mg/kg group versus control group and 2 mg/kg group, resp.; d, e, and f: *P* < 0.0001, *P* < 0.0001, and *P* < 0.001 in 6 mg/kg group versus control, 2 mg/kg, and 4 mg/kg groups, resp.).

**Figure 2:**
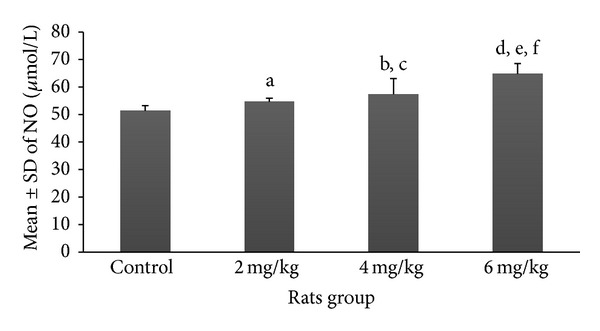
Prenatal exposure to nicotine in pregnant rats increased inflammatory marker of IL-6 in newborn rats. Mean ± SD of the IL-6 marker is shown in different groups of newborn rats. *N* = 8 (a: *P* > 0.05 in 2 mg/kg group versus control group; b and c: *P* < 0.001 and *P* > 0.05 in 4 mg/kg group versus control group and 2 mg/kg group, resp.; d, e, and f: *P* < 0.0001, *P* < 0.001, and *P* < 0.001 in 6 mg/kg group compared to control, 2 mg/kg, and 4 mg/kg groups, resp.).

**Figure 3:**
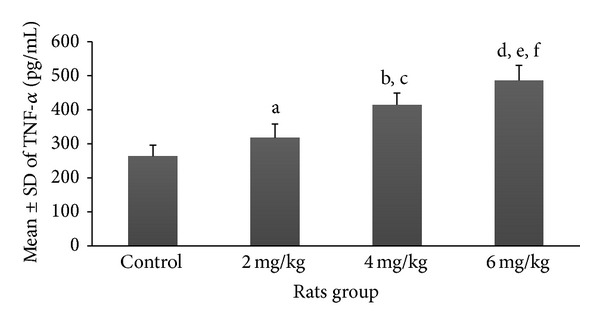
Prenatal exposure to nicotine in pregnant rats increased the inflammatory marker TNF-*α* in newborn rats. Figure shows mean ± SD of the TNF-*α* marker in different groups of newborn rats. *N* = 8 (a: *P* > 0.05 in 2 mg/kg group versus control group; b and c: *P* < 0.001 and *P* > 0.05 in 4 mg/kg group versus control group and 2 mg/kg group, resp.; d, e, and f: *P* < 0.0001, *P* < 0.0001, and *P* < 0.05 in 6 mg/kg group compared to control, 2 mg/kg, and 4 mg/kg groups, resp.).

**Figure 4:**
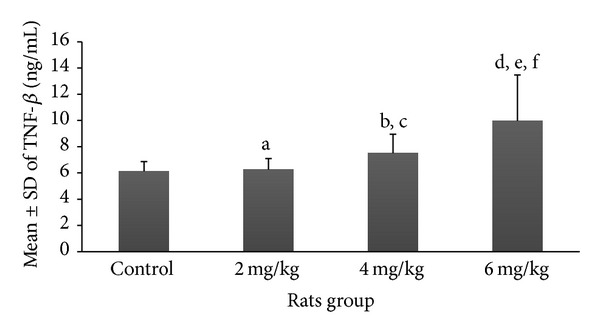
Prenatal exposure to nicotine in pregnant rat increased inflammatory marker TGF-*β* in newborn rats. Mean ± SD of the TGF-*β* marker is shown in different groups of newborn rats. *N* = 8 (a: *P* > 0.05 in 2 mg/kg group versus control group; b and c: *P* < 0.05 and *P* > 0.05 in 4 mg/kg group versus control group and 2 mg/kg group, resp.; d, e, and f: *P* < 0.0001, *P* < 0.0001, and *P* < 0.001 in 6 mg/kg group compared to control, 2 mg/kg, and 4 mg/kg groups, resp.).

**Figure 5:**
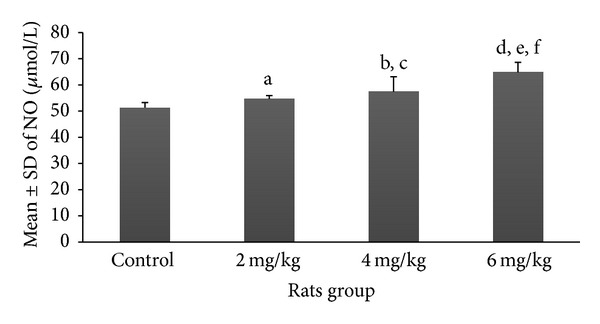
Prenatal exposure to nicotine in pregnant rat increased inflammatory marker nitrite oxide in newborn rats. Mean ± SD of the NO marker in different groups of newborn rats. *N* = 8 (a: *P* > 0.05 in 2 mg/kg group versus control group; b and c: *P* < 0.05 and *P* > 0.05 in 4 mg/kg group versus control group and 2 mg/kg group, resp.; d, e, and f: *P* < 0.001, *P* < 0.001, and *P* < 0.05 in 6 mg/kg group compared to control, 2 mg/kg, and 4 mg/kg groups, resp.).

Mean and SD of various serum markers among rats pinkies exposed to the different dosages of nicotine are demonstrated in Figures 1–5. Each exposure group was compared to control group and to other exposure groups and their relevant *P* values are indicated as figure legends.

According to the results of this study mean ± SD of hs-CRP was significantly higher among groups exposed to various dosages of nicotine (2 mg/kg (316.04 ± 50.2 ng/mL), 4 mg/kg (427.3 ± 49.8 ng/mL), and 6 mg/kg (527.3 ± 35.3 ng/mL)) compared to the control group (262.5 ± 17.5 ng/mL) (*P* < 0.05, *P* < 0.001, *P* < 0.001, resp.) and its increasing rate was also dose dependent (Figure 1). The results of comparison between different exposure groups for hs-CRP are shown as figure legends.

Mean ± SD of IL-6 among different exposed groups to nicotine including 2 mg/kg (39.4 ± 5.4 pg/mL), 4 mg/kg (43.2 ± 3.7 pg/mL), and 6 mg/kg (52.5 ± 4.3 pg/mL) increased compared to the control group (34.4 ± 7.5 pg/mL) (*P* = 0.07, *P* < 0.001, and *P* < 0.001, resp.) and showed a dose dependent trend; however, the difference between group exposed to 2 mg/kg nicotine compared to the control group was not significant (*P* = 0.07). *P* values for difference between groups exposed to 4 and 6 mg/kg nicotine compared to the control group were significant and its increasing rate was dose dependent (Figure 2). The results of comparison between different exposure groups for IL-6 are shown as figure legends.

Mean ± SD of TNF-*α* was considerably increased among groups exposed to the dosages of 4 mg/kg (414.3 ± 34.7 pg/mL) and 6 mg/kg nicotine (485.8 ± 44.5 pg/mL) compared to the control group (263.3 ± 32.6 pg/mL) with significant differences (*P* < 0.05, *P* < 0.001, resp.); however, comparison between the group exposed to 2 mg/kg nicotine (317.4 ± 40.6 pg/mL) and the control group did not show a significant difference (Figure 3). The results of comparison between different exposure groups for TNF-*α* are shown as figure legends.

Mean ± SD of TGF-*β* among the group exposed to 2 mg/kg of nicotine (6.25 ± 0.89 ng/mL) compared to the control group (6.1 ± 0.7 pg/mL) did not show a significant difference; however, there was a significant difference in nicotine exposure groups of 4 mg/kg (7.5 ± 1.4 ng/mL) and 6 mg/kg (9.9 ± 1.4 ng/mL) compared to the control group (*P* < 0.05, *P* < 0.001, resp.) (Figure 4). The results of comparison between different exposure groups for TGF-*β* are shown as figure legends.

Though increasing the dosages of nicotine showed a mild increase in the mean ± SD serum levels of nitrite oxide among nicotine exposure groups of 2 mg/kg (54.7 ± 1.3 *μ*mol/L) and 4 mg/kg (57.4 ± 5.7 *μ*mol/L), only the group exposed to 6 mg/kg of nicotine (64.8 ± 3.8 *μ*mol/L) showed a significant difference (*P* < 0.001) in comparison with the control group (51.3 ± 1.99 *μ*mol/L) (Figure 5). The results of comparison between different exposure groups for nitrite oxide are shown as figure legends.

## 5. Discussion

The present study was launched to detect the inflammatory effects of nicotine on rat embryo by exposing their mothers to different dosages of nicotine during pregnancy. Investigated serum markers were TGF-*β*, TNF-*α*, IL-6, hs-CRP, and nitrite oxide. All these markers were affected by nicotine significantly. The results of this study also showed a dose dependent trend for almost all the studied serum markers among the exposed groups. For instance, increasing the dosages of nicotine during pregnancy significantly increased the above mentioned serum markers. Many diseases are associated with disturbances of these serum markers and their disturbances are also the precursors of some autoimmune and organic diseases. Vascular diseases are usually accompanied with disturbances of inflammatory markers such as Leptin, IL-6, CRP, TNF-*α*, and E-selectin [18, 20–22]. Leptin, IL-6, and CRP via deduction of nitrite oxide affect the endothelial activities and this process causes vascular contraction, leucocytic adherence, platelets activation, oxidative stress, and thrombosis that will result in endothelial dysfunction. These disturbances will be accompanied later with atherosclerosis [23–25]. Some studies reported that smoking during pregnancy causes variations in the activities of immunologic systems of newborns and it also causes a restraint of the anti-inflammatory markers such as IL-10 and a rise in the preinflammatory markers such as TNF-*α*, IL-6, IL-1b, IL-8, and GM-CSF [26]. According to a study by Gaun and others nicotine can change the perivascular fat and affect the vascular contractions causing hypertension [15]. There are different studies to report the mechanism and pathologic effects of nicotine on cardiac development and its activities during pregnancy. A study by Negi and colleagues by an experimental study reported that prenatal application of nicotine causes arterial hypertension of embryo, reduction of oxygen saturation of the blood, and reduction of palpitation in the embryo [17]. Another study by Baykan and coworkers reported that exposing pregnant rats to nicotine caused a reduction of the antioxidant enzymes of myocardium and a rise in the production of peroxides lipids and free radicals of their newborns [16]. Also exposing pregnant rats to nicotine caused an increase in the secretion of sympathetic neurotransmitters of their newborns and, by suppressing the PKC gene, caused rising of cardiac complications in their embryo [4]. Some other studies have reported the effects of prenatal exposure of rats to nicotine and its associated effects on cardiovascular system such as tachycardia, arrhythmia, ischemia, and atherosclerosis [27–30]. Totally, all nicotine exposed groups showed disturbances of the serum levels of all the evaluated inflammatory markers in comparison with the nonexposed group and a dose dependent trend was seen among almost all the evaluated markers in exposed rats. In accordance with the results of our study, almost all reports in this field have confirmed that maternal smoking or prenatal nicotine application shows deleterious effects or serologic disturbances on their embryo.

## 6. Conclusion

The current study showed that exposing pregnant rats to nicotine causes a dose dependent increasing trend for all the studied inflammatory serum markers among their newborns. This phenomenon can result in some complications and diseases associated with these serologic disturbances.

## Figures and Tables

**Table 1:** General characteristics of rats and their newborns in 4 different groups.

Variable (*n*)	Control (8)	2 mg/kg/day (8)	4 mg/kg/day (8)	6 mg/kg/day (8)
Pregnancy duration (hrs)	541.8 ± 0.84	542.3 ± 0.65	542.4 ± 1.04	545.3 ± 1.5^a,b,c^
Birth weight (gr)	7.7 ± 0.26	7.3 ± 0.2^a^	6.6 ± 0.2^a,b^	5.4 ± 0.3^a,b,c^
Total weight gain (gr)	181.4 ± 4.4	171.46 ± 3.5^a^	167.1 ± 3.1^a^	156.9 ± 1.5^a,b,c^
Gender (male %)	54	45	46	47

^a^ *P* < 0.05 compared to control group.

^b^ *P* < 0.05 compared to 2 mg/kg/day group.

^c^ *P* < 0.05 compared to 4 mg/kg/day group.
